# Sweet’s Syndrome Unveiling a Primary Cervical Malignancy: A Case Report

**DOI:** 10.7759/cureus.70541

**Published:** 2024-09-30

**Authors:** Chiranjita Phukan, Saptadweep Saha, Bhaskar J Sarma, Madhumita P Das, Manashi Barman

**Affiliations:** 1 Internal Medicine, Gauhati Medical College and Hospital, Guwahati, IND; 2 College of Medicine, Gauhati Medical College and Hospital, Guwahati, IND

**Keywords:** cervical cancer, elevated inflammatory markers, erythematous papules, fever with rash, malignancy associated sweet syndrome, neutrophilic dermatosis, primary cervical malignancy, rare dermatological condition, skin biopsy, sweet’s syndrome

## Abstract

Sweet's syndrome (SS) is a relatively rare dermatological condition typically presenting with erythematous tender plaques, papules, nodules, or pustules accompanied by fever, asymmetric polyarthralgia, and other systemic features. Elevated inflammatory markers and white blood cell counts are also commonly seen in SS. Dense neutrophilic infiltration in the dermis is a cardinal feature of SS.

A 52-year-old woman presented with a five-month-long history of fever, easy fatigability, multiple painful skin lesions over her scalp, face, trunk, and limbs, and a history of multiple joint pain for three months. Examination revealed tender, erythematous papules, plaques, and nodules which spread from her scalp to involve almost the entire body.

Hematological workup unmasked an overlap between anemia of chronic disease and autoimmune hemolytic anemia. Her inflammatory markers and lactate dehydrogenase (LDH) were also elevated. The anti-nuclear antibody (ANA) test revealed 1:100 titers but the ANA profile failed to point toward a concrete rheumatological cause. After ruling out more common causes of fever with rash, a skin biopsy helped us to diagnose SS conclusively.

A diagnosis of SS necessitated a hunt for the cause. Ultrasonography of the abdomen alarmed us to a suspicious cervical mass which was confirmed as a FIGO (International Federation of Gynecology and Obstetrics) grade IIb neoplasia by an MRI of the pelvis. Histopathological examination established a diagnosis of moderately differentiated squamous cell carcinoma. SS was successfully treated with colchicine.

Our case is unique in the sense that SS was the first feature of a newly diagnosed case of cervical cancer which is already an exceptionally rare cause of SS. There ought to be a greater awareness regarding both SS and its association with malignancy. It’s pertinent that we look out for cancer when more common causes of SS do not fit the clinical picture.

## Introduction

Sweet's syndrome (SS), also known as acute febrile neutrophilic dermatosis, is characterized by a sudden onset of fever, leukocytosis, and the appearance of erythematous plaques and nodules infiltrated by neutrophils. There are three main forms of SS: classical, malignancy-associated, and drug-induced [1]. It may be the initial indication of a relapse of cancer or the skin manifestation of an undetected cancer. One in four SS cases is associated with cancer, of which 85% have underlying hematopoietic neoplasia, most commonly, acute myeloid leukemia (AML) [2]. Other less frequently associated malignancies reported are of genitourinary organs, breast, and gastrointestinal tract, frequently as adenocarcinomas. Cervical cancer is an extremely rare cause of malignancy-associated SS [3].

## Case presentation

**Figure 1:**
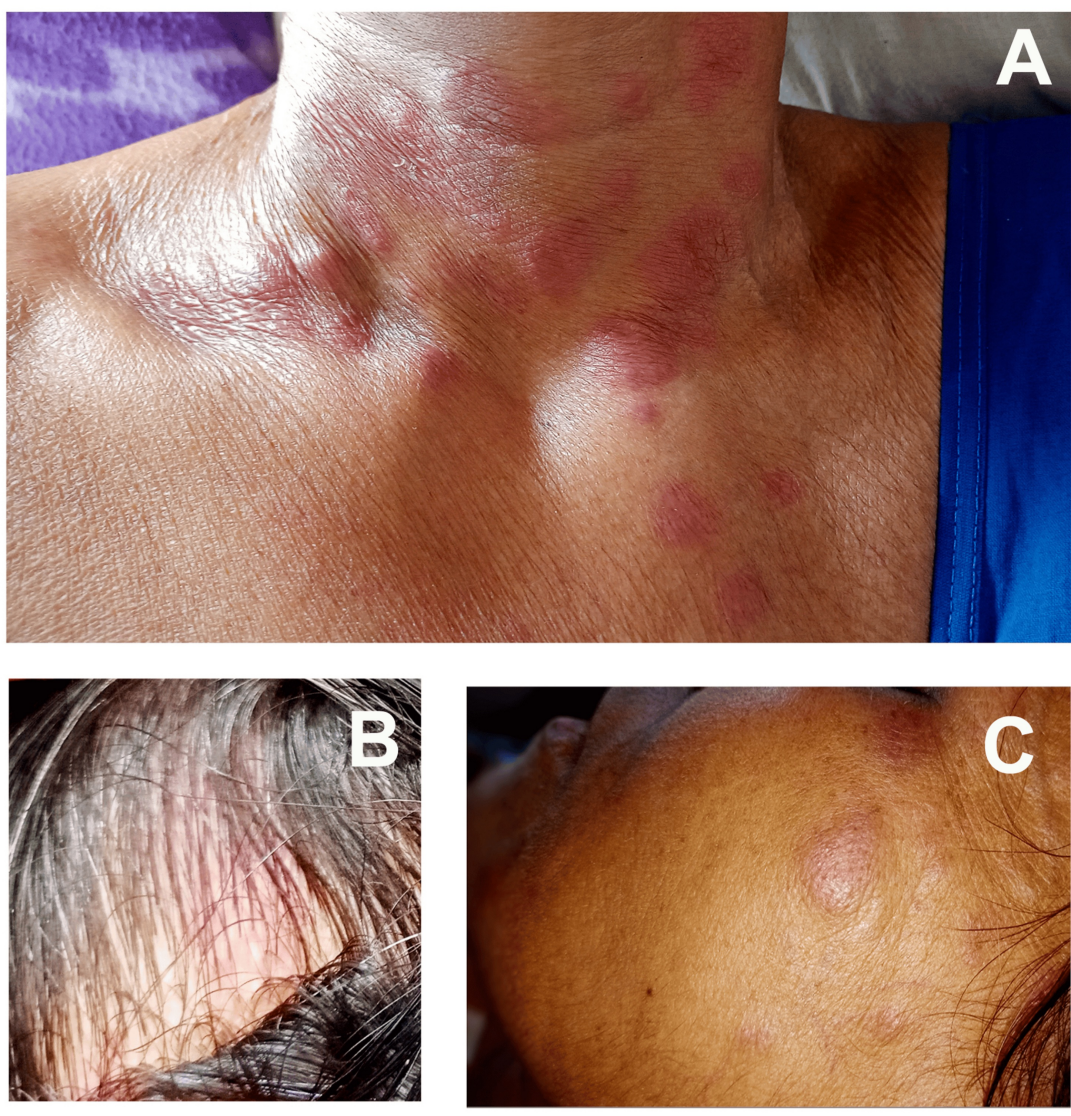
Images showing erythematous rash affecting the chest and neck (A), scalp (B), and face (C).

A 52-year-old Indian female presented with intermittent fever, multiple painful skin lesions for five months, and pain in both large and small joints of both upper and lower limbs for three months. She also reported weight loss and fatigue. Her past medical history is unremarkable. Apart from pallor, the physical examination revealed multiple tender, erythematous papules, plaques, and a few nodules ranging from 2 to 5 cm in size. These lesions initially appeared on the scalp before spreading to the face, neck, trunk, and limbs (Figures 1A-1C). There was no itching, burning sensation, or loss of sensation over the lesions. The ulnar and common peroneal nerves were bilaterally palpable without tingling, numbness, or decreased sensation. The large and small joints of the upper and lower limbs were tender but no swelling, redness, or warmth, and morning stiffness was noted, indicating an asymmetric polyarthralgia pattern of joint involvement.

On admission, her temperature was recorded at 102°F, with a heart rate of 110 beats per minute, blood pressure of 110/74 mmHg, and a respiratory rate of 18 breaths per minute. A complete blood count unmasked moderate anemia with hemoglobin at 7.5 gm/dL. The white blood cell count was fairly normal with 9010 cells/uL; the differential leukocyte count revealed 75.5% neutrophils, 19.2% lymphocytes, 2.7% monocytes, and 2.6% eosinophils.

Mean corpuscular volume (MCV), mean corpuscular hemoglobin (MCH) and mean corpuscular hemoglobin concentration (MCHC) were all decreased revealing a microcytic hypochromic picture which was confirmed by a peripheral blood smear. The inflammatory markers were elevated (erythrocyte sedimentation rate (ESR): 132mm/hr and C-reactive protein (CRP): 23.7 mg/dL) indicating an ongoing severe infection or inflammation or tissue damage. Hemoglobin levels of the patient dropped down to 4.4 gm/dL within a matter of seven days. The corrected reticulocyte index of the patient was calculated to be 0.81 which was consistent with the picture of hypoproliferative anemia with a microcytic hypochromic pattern. Serum iron (30 µg/dL) and total iron binding capacity (162.0 µg/dL) were decreased. Serum ferritin levels were normal (196 ng/mL).

The mosaic of low serum iron levels with serum ferritin >12 ng/mL, elevated inflammatory markers, and transferrin saturation of more than 15% perfectly delineated the picture of anemia of chronic disease [4]. The rapid drop in hemoglobin with a positive Coombs test and highly elevated lactate dehydrogenase (LDH) (1499 U/L) suggests a possible overlap with autoimmune hemolytic anemia. Her alanine aminotransferase and aspartate aminotransferase levels were also elevated, i.e., 409 U/L and 344 U/L, respectively with normal bilirubin levels. Elevated liver enzymes have been noted in previous cases of SS [5].

Anti-streptolysin-O (ASO) titers, rheumatoid factor, anti-cyclic citrullinated peptide (CCP), perinuclear anti-neutrophil cytoplasmic antibodies (pANCA), and cytoplasmic anti-neutrophil cytoplasmic antibody (cANCA) were normal. A slit skin smear for lepra bacilli returned negative results. Anti-nuclear antibody (ANA) screening test was positive with antibody titers of 1:100 dilution with a speckled pattern. ANA profiling revealed positive results for anti-nucleosome, anti-ribonucleoprotein (RNP) 68kD/A/C, anti-Smith/RNP, circulating anticentromere (CENP)-A/B, and sp100 autoantibodies. Equivocal responses were observed for dsDNA, histones, and Sm antibodies. Serum C3 and C4 levels of the patient were within normal limits. A 24-hour urinary protein test revealed proteinuria of 400 mg/day. The investigation findings are summarized in Tables 1-2.

**Table 1:** Summary of investigations conducted to determine the type of anemia.

S. No.	Parameters	Values	Reference Ranges
1.	Haemoglobin on 24/05/24	7.5 g/dL	12-16 g/dL (in females)
2.	Haemoglobin on 31/05/24	4.4 g/dL	12-16 g/dL (in females)
3.	Mean corpuscular volume (MCV)	72.6 fL	76-98 fL
4.	Mean corpuscular hemoglobin (MCH)	20.5 pg	27-32 pg
5.	Mean corpuscular hemoglobin concentration (MCHC)	28.3 g/dL	32-36 g/dL
6.	Serum iron	30 µg/dL	49-181 µg/dL
7.	Total iron binding capacity (TIBC)	162 µg/dL	261-462 µg/dL
8.	Transferrin saturation	18.5%	15-50% (in females)
9.	Serum ferritin	196 ng/mL	10-150 ng/mL
10.	Lactate dehydrogenase (LDH)	1499 U/L	120-246 U/L
11.	Erythrocyte sedimentation rate (ESR)	132 mm/hr	6-20 mm/hr
12.	C-reactive protein (CRP)	23.7 mg/dL	0-10 mg/dL
13.	Direct and indirect Coombs test	Positive	Not applicable

**Table 2:** Summary of other relevant investigations.

S. No.	Parameters	Values	Reference Ranges
1.	Complete blood count (CBC)	9010 cells/µL	4000-11000 cells/µL
2.	Alanine aminotransferase (ALT)	409 U/L	4-36 U/L
3.	Aspartate aminotransferase (AST)	344 U/L	0-35 U/L
4.	Rheumatoid factor (RF)	Negative	<10 IU/mL
5.	Anti-streptolysin-O titers (ASO)	Negative	<200 IU/mL
6.	Perinuclear anti-neutrophil cytoplasmic antibody (pANCA)	3.1 AU/mL	Negative: <19 AU/mL
7.	Cytoplasmic anti-neutrophil cytoplasmic antibody (cANCA)	2 AU/mL	Negative: <19 AU/mL
8.	Anti-cyclic citrullinated peptide (anti-CCP)	Negative	Negative: <5 U/mL
9.	Anti-nuclear antibody (ANA)	1: 100 antibody titre	Not applicable
10.	Serum C3	92 mg/dL	88-165 mg/dL
11.	Serum C4	23.80 mg/dL	14-44 mg/dL
12.	24-hr urinary protein	400 mg/day	<150 mg/day

The differential diagnosis for fever with rash and arthralgia includes autoimmune and autoinflammatory rheumatic diseases as well as infectious diseases which were ruled out based on key clinicopathological features as discussed below. Erythema nodosum was ruled out as the lesions were not bilaterally symmetrical and did not involve the shin. Histopathological examination definitively ruled it out. Although the ANA titer in this patient was 1:100, however, the clinical and laboratory findings did not fulfill the EULAR/ACR (European League Against Rheumatism/American College of Rheumatology) 2019 criteria making systemic lupus erythematosus (SLE) unlikely. The lack of typical palpable purpura (small vessel vasculitis) or nodular rash (medium vessel vasculitis), along with normal pANCA and cANCA levels, excluded vasculitis. Leukemia cutis was excluded by the absence of neoplastic immature leukocytes on skin biopsy. Infectious causes, such as viral diseases like chikungunya, bacterial infections like Lyme disease and tuberculosis, as well as reactive arthritis that may develop weeks after infection, can lead to fever, rash, and arthritis. However, the patient presented with polyarthralgia and showed no features of inflammatory arthritis, thereby excluding these potential causes.

**Figure 2:**
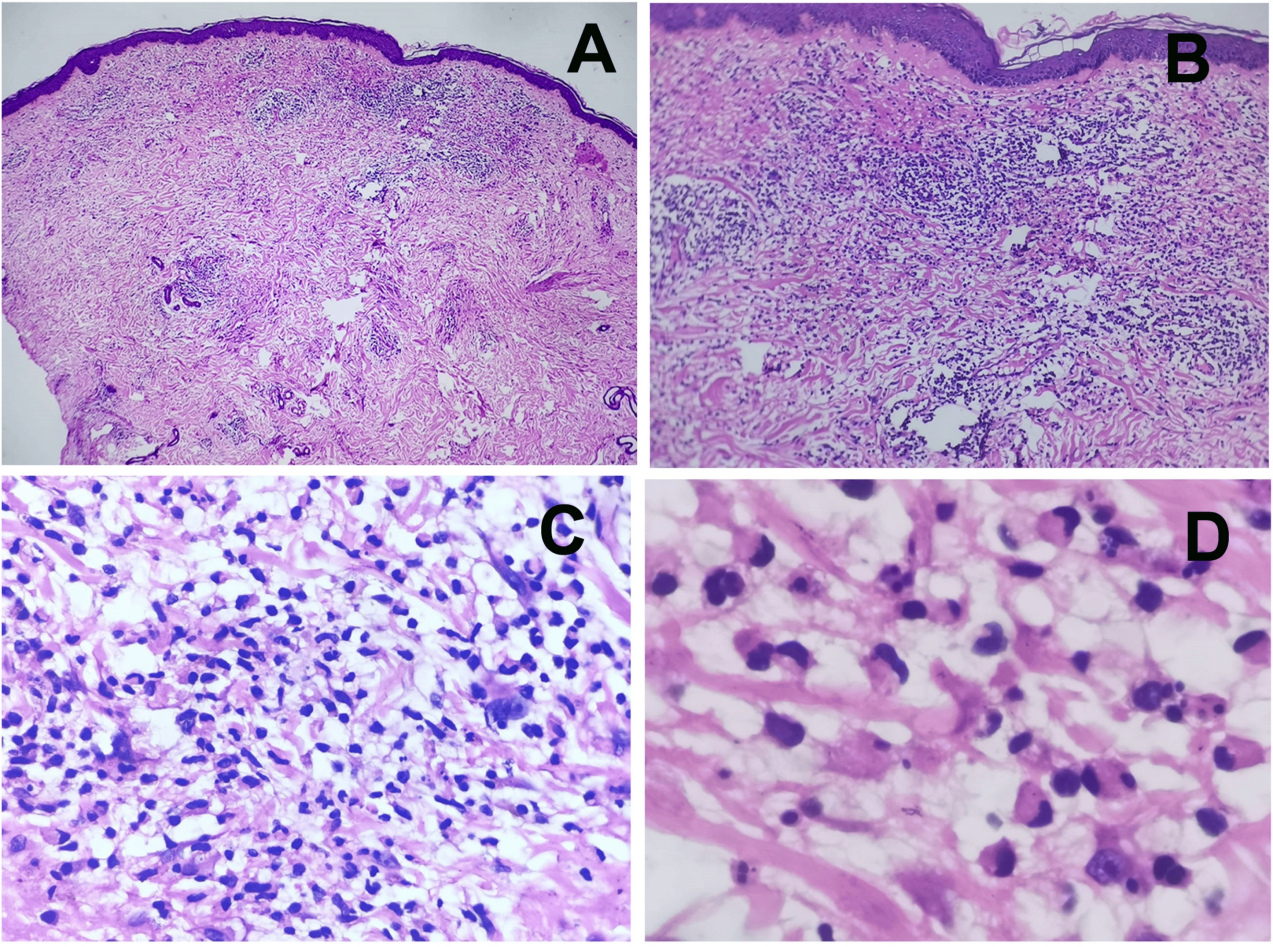
Histopathology of the skin punch biopsy from the lesional area. Serial sections reveal skin with an unremarkable epidermis. There is evidence of papillary dermal edema, while the superficial and reticular dermis show a dense cellular infiltrate comprising neutrophils with leukocytoclasia, mixed with lymphocytes. Additionally, numerous histiocyte-like cells with reniform nuclei and scant eosinophilic cytoplasm are present. (A: 10x, B: 40x, C: 100x, D: 400x magnification; H & E stain)

A punch biopsy taken from a skin lesion on the chest revealed a “dermal cellular infiltrate comprising neutrophils with leukocytoclasia, mixed with lymphocytes and numerous histiocyte-like cells within the infiltrate,” clinching the diagnosis of neutrophilic dermatosis, favoring histiocytoid SS. The histopathological images as seen under the microscope are shown in Figures 2A-2D [6].

**Figure 3:**
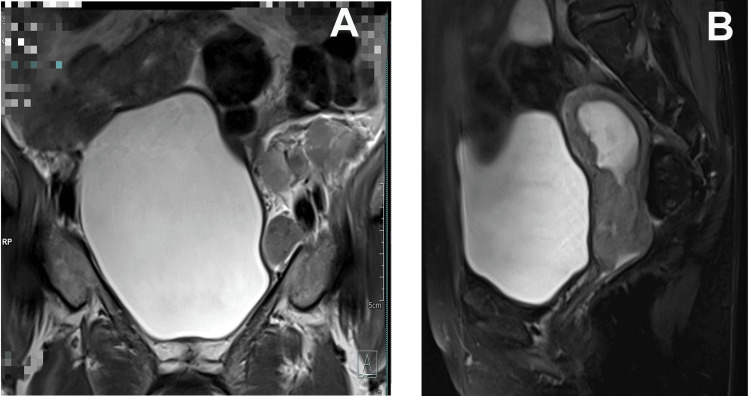
MRI of the pelvis showing (A) a coronal section and (B) a sagittal section. MRI pelvis (A): Coronal section showing metastatic lymphadenopathy in the left external iliac and left obturator stations, forming a nodal mass that abuts the left common and external iliac vessels. Involvement of the right external iliac and obturator stations is also noted. MRI pelvis (B): Sagittal T2 fat-saturated image revealing an irregular circumferential mass lesion involving both the anterior and posterior lips of the cervix, with narrowing of the endocervical canal.

Ultrasonography of the whole abdomen displayed "an ill-defined heterogeneously hyperechoic lesion in the cervical region with stenosis of distal part with endometrial collection with locoregional and distant lymphadenopathy suspicious of cervical cancer.” A gynecological history and evaluation revealed that her last menses was four months ago with no history of post-menopausal bleeding. With a parity of six, she has three living children. A flushed and pulled-up cervix was found during speculum inspection, along with a tiny amount of slightly purulent discharge. The MRI pelvis revealed "irregular circumferential cervical mass lesions with extension to lower body of uterus and involvement of vaginal fornices, parametrial invasion, and locoregional metastatic lymphadenopathy” suggestive of neoplastic etiology of FIGO (The International Federation of Gynecology and Obstetrics) grade IIb (Figures 3a, 3b). Histopathological examination of the cervical tissue revealed moderately differentiated squamous cell carcinoma.

Initially, she was treated with oral prednisolone 30 mg once daily with limited success. Upon diagnosis of cancer cervix, we switched to colchicine 1.5 mg/day in three divided doses. The cardinal complaints of fever accompanied by rashes and joint pain decreased significantly in two days and resolved completely in three weeks. The skin lesions healed without scarring but left hyperpigmented patches which slowly disappeared. She was referred to the Department of Surgical Oncology for appropriate management of squamous cell carcinoma of the cervix.

## Discussion

The diagnosis of SS is established based on the presence of two major criteria and any two of the three minor criteria. The major criteria include (1) an abrupt onset of painful erythematous plaques or nodules and (2) a dense neutrophilic infiltrate on histopathology, which shows no evidence of leukocytoclastic vasculitis. The minor criteria consist of (1) fever greater than 38°C, (2) association with an underlying hematologic or visceral malignancy, inflammatory disease, or pregnancy, or a history of upper respiratory or gastrointestinal infection or vaccination preceding the onset. (3) An excellent response to treatment with systemic corticosteroids or potassium iodide is noted. (4) Laboratory findings may also show abnormalities at presentation (three of four), including an ESR greater than 20 mm/hour, positive CRP, leukocyte counts exceeding 8,000, and neutrophil percentages greater than 70% [1].

The erythematous plaques, papules, and nodules seen here are classic for SS. Patients often present acutely ill, with fever being the most frequent symptom. Arthralgia, as seen in this case, is the second most common systemic manifestation [7]. Elevated ESR, CRP, and white blood cell counts match the diagnostic criteria for SS. The absence of an associated history of gastrointestinal or respiratory infection or vaccination makes malignancy the most likely cause of SS.

Renal involvement, as demonstrated by proteinuria here, is another extremely uncommon presentation seen in SS [5]. SS is also known to be caused by several drugs such as cotrimoxazole, mercaptopurine, and azathioprine. However, our patient had no history of intake of such drugs [8]. Recent studies have linked raised ANA titers with speckled patterns to invasive cervical cancer, a possible explanation for ANA positivity in this case [9]. It has been suggested that circulating autoantibodies, cytokines, immunological complexes, and human leukocyte antigen (HLA) serotypes are involved in the pathophysiology of SS [5].

Corticosteroids are the preferred agents for SS [5]. Out of an abundance of caution, we switched to colchicine because of perceived concerns surrounding the use of steroids in cervical cancer as highlighted in this study [10]. Poor outcomes and recurrence of cervical cancer have been associated with SS [11]. However, in our patient, SS was the presenting feature of primary cervical malignancy.

## Conclusions

Greater awareness of SS as a possible sign of solid organ cancer can help clinicians detect cancer early and improve patient outcomes. In our case, it was an inexpensive ultrasound of the abdomen that provided a clue to the cause. Since the entire diagnosis hinges on histopathology, it is pertinent that SS does not elude the pathologist’s eye. The bizarre ANA profile in this case calls for further research into the underlying immune response in malignancy-associated SS. To the best of our knowledge, this is the first reported case where SS was the earliest sign of cervical cancer.
